# Clinical profile of acute myocardial infarction: the Registry of Acute Medical Emergencies registry

**DOI:** 10.1590/1806-9282.20251775

**Published:** 2026-08-10

**Authors:** Pedro Gabriel Melo de Barros e Silva, Alexandre Matos Soeiro, Rodrigo Balada, Lucas Macedo, Milena Del Valle de Lacerda, Luan Vitor Aguiar Correia, Maria Carolina Ximenes, Karen Teixeira Dias, Sabrina Carvalho Moura, Sofia Gabriella Gregolini Catellani, Renato Deslacio Lopes

**Affiliations:** 1 Hospital Samaritano Paulista – São Paulo (SP), Brazil.; 2 Brazilian Clinical Research Institute – São Paulo (SP), Brazil.; 3 Centro Universitário São Camilo – São Paulo (SP), Brazil.; 4 Universidade de São Paulo, Heart Institute – São Paulo (SP), Brazil.; 5 Duke University, Duke Clinical Research Institute – Durham (NC), United States.

**Keywords:** Acute coronary syndromes, Chest pain, Signs and symptoms, ST elevation myocardial infarction

## Abstract

**INTRODUCTION:**

Around 30% of patients with acute myocardial infarction do not report chest pain, but data on symptom patterns according to the type of myocardial infarction and attending physician specialty are scarce.

**OBJECTIVE:**

The aim of this study was to identify common clinical presentations of acute myocardial infarction in a Brazilian registry, stratified by infarction type and physician specialty.

**METHODS:**

Observational analysis of two datasets: (1) retrospective cohort of 2,783 acute myocardial infarction patients from 15 hospitals (2014–2018) initially evaluated by emergency physicians; (2) prospective registry of 718 acute myocardial infarction patients (2018–2020) assessed in the acute phase by cardiologists via telemedicine. Cases were classified as ST-segment Elevation Myocardial Infarction or Non-ST-segment Elevation Myocardial Infarction. Symptoms were categorized as typical/atypical chest pain, epigastric pain, or absence of both; additional symptoms were recorded. Categorical variables were compared using chi-square tests (p<0.05).

**RESULTS:**

Typical chest pain occurred in 60.3% of patients, more frequent in ST-segment Elevation Myocardial Infarction than Non-ST-segment Elevation Myocardial Infarction (66.5 vs. 56.5%; p<0.01). Absence of chest/epigastric pain was more common in Non-ST-segment Elevation Myocardial Infarction (6.1%) than ST-segment Elevation Myocardial Infarction (2.7%) (p<0.01). Cardiologist assessment identified more acute myocardial infarction cases without chest/epigastric pain than emergency physician assessment (11.0 vs. 3.2%; p<0.01). ST-segment Elevation Myocardial Infarction patients without chest/epigastric pain had the highest mortality (29.4%) in the emergency physician cohort.

**CONCLUSION:**

In this registry, absence of chest or epigastric pain was less frequent than previously reported but associated with high mortality. Telemedicine evaluation by cardiologists identified a greater proportion of pain-free acute myocardial infarction presentations.

**Clinical Trial Registration Number:**

ClinicalTrials.gov NCT02753023.

## INTRODUCTION

Almost one third of deaths worldwide are due to cardiovascular disease, and ischemic cardiac disease represent the main cause of cardiovascular death^1^. Reduction on the mortality due to acute myocardial infarction (AMI) depends on appropriate and fast diagnosis of this condition^2,3^. For decades, the protocol developed to guide the diagnosis and treatment of patients with suspected AMI is commonly denominated as “chest pain protocol” due to the high frequency of this symptom among patients with AMI^4^. However, almost one third of the patients with acute coronary syndrome do not report chest pain in the admission^5^. Focusing the investigation only on patients with chest pain may lead to a lower or later recognition of a numerically relevant group of AMI patients. In addition, these patients without chest pain presented worse clinical outcomes in previous studies, which reinforces the need of expanding these protocols to also investigate patients without chest pain^5,6,7^. Nevertheless, the variety of symptoms that could be related to AMI could make difficult to implement more comprehensive inclusion criteria in chest pain protocols at many sites.

Contemporary data regarding symptoms according to type of AMI (with or without ST elevation) is limited and this discrimination would be helpful for sites that local resources are limited and need to prioritize chest pain protocol inclusion for cases of suspicion of ST-elevation myocardial infarction (STEMI).

In addition, information about recognition of symptoms according to medical specialty are also scarce, especially in Latin American populations. Since physicians with different backgrounds commonly work in emergency departments in Latin America, variations in recognition may lead to variations in medical care.

The objective of the current study is to identify, in a Brazilian registry, the most common types of clinical presentation among patients with myocardial infarction (MI) and correlate with in-hospital mortality. This analysis will explore the overall population of MI patients and also according to the type of MI (STEMI or not) and to the type of medical evaluation in the acute phase (with or without support of a cardiologist by telemedicine).

## METHODS

### Study design

ROAD (Registry of Acute Medical Emergencies) is a registry that collects data of consecutive patients presenting to participating hospitals with different acute medical conditions, including cases of AMI. The current analysis explored patients with AMI included by a private medical network which integrates the ROAD registry.

### Study population

Overall, the current analysis included 3,602 consecutive patients with AMI that were categorized in two groups according to the medical evaluation in the acute phase: (1) Emergency physician assessment: 2,884 patients with AMI from 15 hospitals, evaluated initially by non-cardiologists, and included from January of 2014 to December of 2018.

(2) Cardiologist assessment in the acute phase: 718 consecutive MI patients from the same hospitals evaluated during the acute phase by cardiologists through a telemedicine network from January 2019 to January 2020 (before pandemic). In both groups, the cases were separated according to the type of MI as STEMI or Non-ST elevation myocardial infarction (NSTEMI). All the cases of myocardial infarction were due to atherothrombotic disease (type 1 MI)^8^.

### Symptoms categories

The initial complaints were categorized as chest pain (typical or atypical), epigastric pain and absence of chest/epigastric pain. The typical or atypical chest pain were based on medical judgment, but the physicians were trained to consider typical pain the cases where the localization was substernal or precordial plus at least one of the following: typical characteristic (pressure, heaviness); typical radiation (arms, jaw); typical factors of relief/worsening (rest or nitrate). Additional symptoms including dyspnea, diaphoresis, and nausea were also registered.

Symptom presentation was recorded according to the clinical assessment documented at the time of first medical contact. In the retrospective cohort (ROAD registry, 2014–2018), symptom information was extracted from emergency department medical records. In the prospective telemedicine registry (2019), symptom classification was recorded in real time by cardiologists during remote consultation. Symptoms were classified into predefined clinical categories at presentation, consistent with routine clinical assessment.

Symptom classification was based on the evaluating physician’s clinical assessment at the time of presentation, reflecting routine clinical practice in the emergency setting.

Symptoms were categorized into predefined clinical groups commonly used in daily care.

### Clinical outcomes

In-hospital mortality is a standard metric assessed by the ROAD registry and includes all cases of death before discharge. This outcome was evaluated separately according to each group analyzed.

### Statistical analysis

Categorical variables were reported as frequencies with percentages, and continuous variables were described by mean and standard deviation. Comparison between groups were made using t-test for continuous variables and chi-square for categorical variables. P-values were two-tailed, and values below 0.05 were considered statistically significant. Analysis was performed using R software, version 3.6.1 (R Foundation for Statistical Computing).

## RESULTS

### Study patients

**Figure 1:**
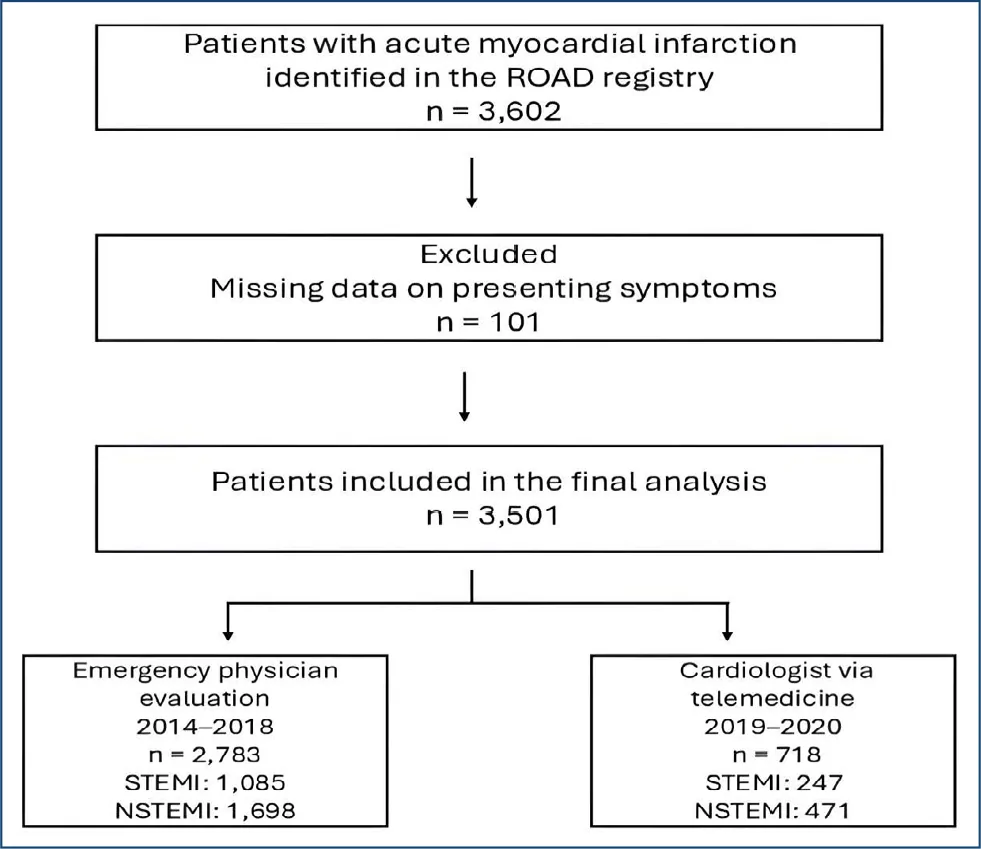
Flowchart of patient selection and study population. A total of 3,602 patients with acute myocardial infarction were identified in the ROAD registry. After exclusion of 101 patients due to missing data on presenting symptoms, 3,501 patients were included in the final analysis and stratified according to evaluation context and myocardial infarction type.

A total of 3,602 consecutive patients with AMI were included in the database and 3,501 were included in the current analysis since 101 patients were excluded due to missing data (Figure 1). Among all patients analyzed, the mean age was 62.5 years and 36.0% were female (Table 1).

**Table 1:** Baseline characteristics and symptoms according to medical evaluation^a,b^.

	Emergency physician assessment (n=2,783)	Cardiologist assessment (n=718)	Overall (n=3,501)
Female %	35.0%	39.8%	36.0%
Mean age in years (±SD)	62.29 (±13.56)	64.51 (±15.17)	62.50 (±13.94)
Hypertension	1,122 (40.3%)	428 (59.6%)	1,550 (44.3%)
Diabetes mellitus	590 (21.2%)	198 (27.6%)	788 (22.5%)
Dyslipidemia	603 (21.7%)	121 (16.8%)	724 (20.7%)
Smoking	436 (15.7%)	76 (10.6%)	512 (14.6%)
Previous history of CAD	322 (11.6%)	41 (5.7%)	363 (10.4%)

^a^SD: standard deviation; CAD: coronary artery disease.

^b^All the symptoms could be associated (e.g., the same patient could have chest pain and dyspnea).

### Overall clinical manifestation

Almost 40% of the 3,501 patients with MI did not have typical chest pain but only 4.8% of all MIs did not have chest or epigastric pain (Table 2).

### Clinical manifestation according to type of myocardial infarction and medical evaluation

**Figure 2:**
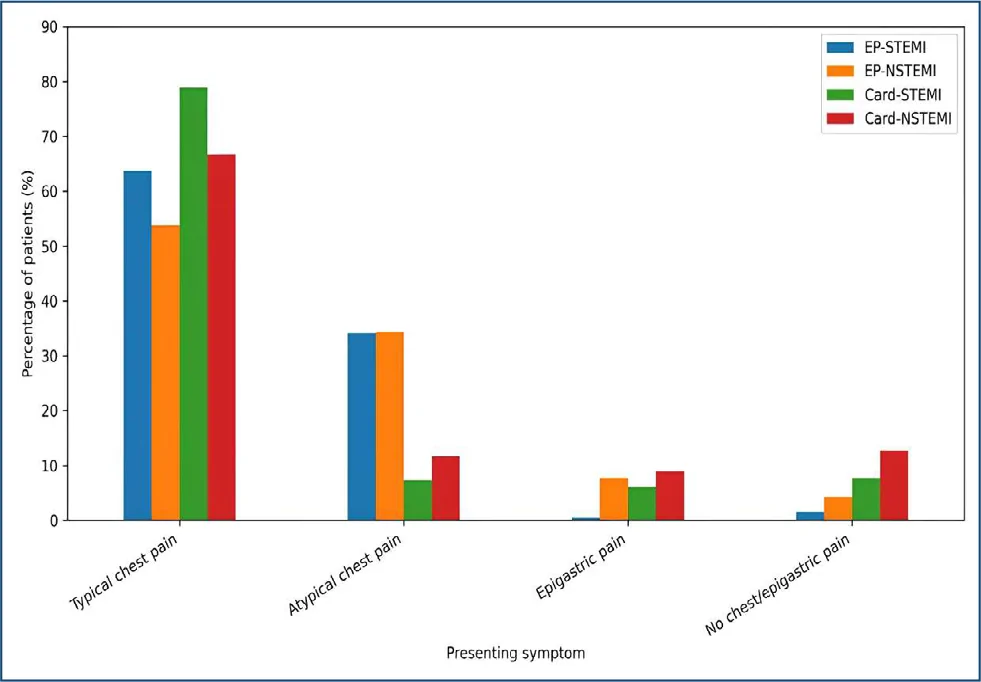
Distribution of presenting symptoms according to myocardial infarction type and evaluation context. Bars represent the percentage of patients presenting with typical chest pain, atypical chest pain, epigastric pain, or absence of chest or epigastric pain, stratified by myocardial infarction type and by evaluation context (emergency physician vs. cardiologist via telemedicine).

In clinical manifestation according to type of MI (Figure 2), there is two information that call attention. First, the prevalence of typical chest pain is 10% higher in STEMI than in NSTEMI (66.5 vs. 56.5%; p<0.01). Second, the prevalence of absent chest pain (epigastric pain or without chest/epigastric) pain is 3.4% higher in NSTEMI than in STEMI (6.1 vs. 2.7%; p<0.01). On the other side, the phase of cardiologist assessment was associated with 15.2% more classification of typical pain in STEMI population and a percentage 8.4% higher of cases of NSTEMI without chest pain than during the period of emergency physicians report (Table 2).

**Table 2:** Presenting symptoms according to myocardial infarction type and medical evaluation context.

Clinical characteristics	EP–STEMI (n=1,085) (38.9%)	EP–NSTEMI (n=1,698) (61.0%)	Card–STEMI (n=247) (34.4%)	Card–NSTEMI (n=471) (65.5%)	Overall (n=3,501)
Typical chest pain	692 (63.7%)	913 (53.7%)	195 (78.9%)	314 (66.6%)	2.114 (60.3%)
Atypical chest pain	371 (34.1%)	582 (34.2%)	18 (7.2%)	55 (11.6%)	1.026 (29.3%)
Epigastric pain	5 (0.4%)	130 (7.6%)	15 (6.0%)	42 (8.9%)	192 (5.4%)
No chest or epigastric pain	17 (1.5%)	73 (4.2%)	19 (7.6%)	60 (12.7%)	169 (4.8%)
Dyspnea	157 (14.4%)	364 (21.4%)	27 (10.9%)	70 (14.8%)	618 (17.6%)
Diaphoresis	329 (30.3%)	367 (21.6%)	41 (16.5%)	37 (7.8%)	774 (22.1%)
Nausea	236 (21.7%)	270 (15.9%)	24 (9.7%)	63 (13.3%)	593 (16.9%)
Vomit	95 (8.7%)	103 (6.0%)	12 (4.8%)	21 (4.4%)	231 (6.5%)
Malaise	96 (8.8%)	122 (7.1%)	7 (2.8%)	19 (4.0%)	244 (6.9%)

STEMI: ST-segment Elevation Myocardial Infarction; NSTEMI: Non-ST-segment Elevation Myocardial Infarction; EP: emergency physician; STEMI: ST-segment elevation myocardial infarction; NSTEMI: non-ST-segment elevation myocardial infarction; Card: cardiologist (via telemedicine).

### Mortality according to the classification of symptoms in the acute phase assessed by emergency physicians

Hospital mortality data was only available for the retrospective cohort assessed by emergency physicians. Considering both groups together (STEMI and NSTEMI), in-hospital mortality increased according to symptoms with the lower rate among typical chest pain (1.3%) and epigastralgy (1.4%), intermediate among patients with atypical chest pain (4.3%) and highest in the cases of MI without chest or epigastric pain (9.0%) (p<0.01).

Hospital mortality analysis was performed exclusively for the retrospective cohort (emergency physician group), as these data were not available for the telemedicine cohort.

### ST-segment Elevation Myocardial Infarction patients without chest or epigastric pain

This was the group with the highest mortality, with an in-hospital mortality rate of 29.4%, although it represented only 2.7% of all STEMI cases. This presentation was more common among elderly patients (mean age: 65.6 years). Compared to STEMI patients with chest or epigastric pain, those without pain more frequently reported non-specific malaise (7.8 vs. 6.8%; p=0.1122), sweating (27.7 vs. 18.0%; p<0.01), vomiting (8.0 vs. 5.3%; p<0.01), and dyspnea (17.0 vs. 13.0%; p<0.01) (Table 3).

**Table 3:** In-hospital mortality according to symptom category.

Symptom category	Overall mortality %	p-value
Typical chest pain	1.3%	<0.01
Atypical chest pain	4.3%	<0.01
Epigastric pain	1.4%	<0.01
No chest or epigastric pain	9.0%	<0.01

p-values refer to comparison across symptom categories.

## DISCUSSION

In the current study we did identify that the classification of clinical manifestation in patients with MI varied according to the type of MI and the type of medical evaluation. Typical chest pain was approximately 10% more frequent in STEMI patients while atypical presentation (including no pain) was more frequent in NSTEMI patients. A higher proportion of myocardial infarction presentations without chest or epigastric pain was observed during the period of cardiologist evaluation via telemedicine. This finding should be interpreted within the context of prospective cardiologist involvement. This clinical presentation represents a prognostic factor related to higher in hospital mortality.

This large Brazilian registry brings some new information to clinical presentation of MI. The first aspect to consider is the lack of information of clinical symptoms of MI in Latin America, especially in a large contemporary population categorized in STEMI and NSTEMI. Previous reports^9,10,11,12,13,14^ where mainly from outside Latin America, occurred several years ago and most of them did not assess symptoms in AMI patients according to ST elevation. An analysis from the National Registry of Myocardial Infarction (NRMI) that included patients from 1994 to 2006^9^ did separate the types of AMI and showed that patients without chest pain were older and this presentation was two times more common in patients without ST elevation. These findings were also confirmed in Asian patients^10^. The comparison with the NRMI should be interpreted with caution due to differences in symptom definitions. While the NRMI reported a 43.6% rate of myocardial infarction without chest pain, the present study classified symptom presentation into predefined clinical categories. When considering both atypical chest pain and absence of chest or epigastric pain together, the overall proportion of non-typical presentations in our cohort becomes more comparable to that reported in prior studies, highlighting the importance of symptom classification definitions when interpreting differences across registries. Regarding the classification of probability of MI according to the type of MI, not only the percentage of chest pain was higher among STEMI patients when compared to NSTEMI patients, but also the frequency of typical symptoms (higher probability of MI diagnosis based on medical judgement).

Beyond differences based on the type of MI, there is limited knowledge about the variation in symptoms classification according to medical specialty. In this study we could see that specialized evaluation through telemedicine classified cases of STEMI more commonly as typical chest pain the cases and identified more cases of NSTEMI without chest pain. Despite the current trend in using cardiac, possible cardiac and noncardiac chest pain instead typical or atypical^15,16^ the current results indicate that differences in the classification of chest pain may be associated with background training and experience and reinforces the potential benefit of “real time” specialized evaluation for these cases to support the diagnosis. In addition, the identification of cases of MI without chest or epigastric pain may be also related to a higher suspicion of a specialist. Finally, the results of the current study may influence chest pain protocols according to the local capabilities. Considering the very low percentage of patients with STEMI and without chest and epigastric pain, the inclusion criteria of chest protocol in regions with limited resources could prioritize the patients with higher probability of STEMI, a time-sensitive condition. On the other side, services that aim to be more sensitive and capture all MIs should include other symptoms beyond chest or epigastric pain. In order to detect STEMI in patients without pain, an electrocardiogram should be performed in patients (especially elderly) with unexplained malaise, sweating, vomiting and/or dyspnea. Despite the low frequency, it is important to detect this group of patients since the mortality is much higher when compared to other clinical presentation, that are more valued. A support of a specialist by telemedicine may be desirable to improve recognition, especially in settings where the physicians are not specialized in emergency care.

While pain-free AMI was associated with higher mortality in the emergency physician cohort, further studies are needed to confirm if this outcome persists when these patients are identified early through specialized telemedicine support.

### Study limitations

The current study is a large observational registry and the information included was based on medical judgment of one physician (first phase emergency physician and second phase, cardiologist by telemedicine). As consequence, the subjectivity was not assessed by inter-observer variability. Nevertheless, all the physicians were educated to classify with the same criteria and the results represent the real pragmatic classification in clinical practice. Other aspect relevant to be mentioned is the absence of information regarding symptoms among patients without MI. This information would be useful for accuracy analysis, but this was not the target of the current analysis and the large number of patients with confirmed MI strength the aim of the study which is the evaluation of symptoms more frequent according to the type of MI. Finally, emergency medicine is a new medical specialty in Brazil and cardiologists commonly work in emergency departments and have large experience identifying patients with MI. Thus, the differences identified in the current study may be smaller in countries where emergency physicians receive a formal training to conditions like acute MI and cardiologists have less experience in emergency care.

We acknowledge the risk of temporal confounding due to the sequential rather than simultaneous enrollment of the cohorts. The higher prevalence of atypical presentations identified in the later cohort (telemedicine) could be partially attributed to an increased clinical awareness of silent AMI in recent years or to more standardized real-time data entry compared to the earlier retrospective chart reviews. Consequently, the impact of time-related variables on the observed differences cannot be entirely ruled out.

Another limitation is the lack of mortality data for the telemedicine cohort. Consequently, the findings regarding higher mortality in patients without chest pain are restricted to the emergency physician cohort and cannot be generalized to the group assessed via telemedicine. This prevents a direct comparison of clinical outcomes between the two assessment models.

Furthermore, the mortality analysis presented in this study is unadjusted for potential confounders. As observed in Table 1, patients in the cardiologist-led cohort were older and had a higher prevalence of comorbidities, such as hypertension and diabetes, which are independent predictors of mortality. Therefore, the higher mortality observed in patients with atypical presentations may be partially driven by these baseline clinical differences rather than the symptom profile alone. Future studies employing multivariate analysis are required to provide a more robust estimate of the independent impact of presenting symptoms on clinical outcomes.

There is also an inherent inconsistency in symptom classification between the two cohorts. While data for the 2014–2018 period were extracted retrospectively from medical records, symptoms in the 2019 telemedicine cohort were recorded in real-time by cardiologists. This difference in data collection methodology may lead to ascertainment bias, as prospective recording by specialists is generally more accurate and detailed. Thus, the higher identification rate of pain-free AMI by cardiologists might reflect superior documentation and clinical scrutiny rather than a true biological difference in patient presentation between the periods.

## CONCLUSION

In a large registry of a contemporary population of patients with acute myocardial infarction in Brazil, cases without chest or epigastric pain were identified less frequently than previously reported by international literature. This group was associated with higher mortality (observed in the emergency physician cohort) and was more frequently identified with cardiologist support via telemedicine. Our findings should help the development of more assertive inclusion criteria in chest pain protocols and also provide insights regarding tools that could be used to improve symptoms recognition.

## Data Availability

The datasets generated and/or analyzed during the current study are available from the corresponding author upon reasonable request.
