# ERRATUM: Clinical profile of acute myocardial infarction: the Registry of Acute Medical Emergencies registry

**DOI:** 10.1590/1806-9282.20251775-ER

**Published:** 2026-10-12

In the manuscript: Silva PGM de B e, Soeiro AM, Balada R, Macedo L, Lacerda MDV de, Correia LVA, et al. Clinical profile of acute myocardial infarction: the Registry of Acute Medical Emergencies registry. Rev Assoc Med Bras. 2026;72(6):e20251775. https://doi.org/10.1590/1806-9282.20251775, on page 1:

**Where it reads:**

Sofia Gabriela Gregolini Catellani

**It should read:**

Sofia Gabriella Gregolini Catellani

Roseli Nomura https://orcid.org/0000-0002-6471-2125

*Editor-in-Chief*

